# A Polyimide Composite-Based Electromagnetic Cantilever Structure for Smart Grid Current Sensing

**DOI:** 10.3390/mi15101189

**Published:** 2024-09-26

**Authors:** Zeynel Guler, Nathan Jackson

**Affiliations:** 1 Department of Mechanical Engineering, University of New Mexico, Albuquerque, NM 87131, USA; 2 Center for High Technology Materials, University of New Mexico, Albuquerque, NM 87106, USA; 3 Nanoscience and Microsystems Engineering, University of New Mexico, Albuquerque, NM 87106, USA

**Keywords:** microelectromechanical systems (MEMS), composite, polyimide, thin films, electromagnetic sensor, energy harvester

## Abstract

Polyimides (PIs) have been extensively used in thin film and micro-electromechanical system (MEMS) processes based on their excellent thermal and mechanical stability and high glass transition temperature. This research explores the development of a novel multilayer and multifunctional polymer composite electro-piezomagnetic device that can function as an energy harvester or sensor for current-carrying wires or magnetic field sensing. The devices consist of four layers of composite materials with a polyimide matrix. The composites have various nanoparticles to alter the functionality of each layer. Nanoparticles of Ag were used to increase the electrical conductivity of polyimide and act as electrodes; lead zirconate titanate was used to make the piezoelectric composite layer; and either neodymium iron boron (NdFeB) or Terfenol-D was used to make the magnetic and magnetostrictive composite layer, which was used as the proof mass. A novel all-polymer multifunctional polyimide composite cantilever was developed to operate at low frequencies. This paper compares the performance of the different magnetic masses, shapes, and concentrations, as well as the development of an all-magnetostrictive device to detect voltage or current changes when coupled to the magnetic field from a current-carrying wire. The PI/PZT cantilever with the PI/NdFeB proof mass demonstrated higher voltage output compared to the PI/Terfenol-D proof mass device. However, the magnetostrictive composite film could be operated without a piezoelectric film based on the Villari effect, which consisted of a single PI-Terfenol-D film. The paper illustrates the potential to develop an all-polymer composite MEMS device capable of acting as a magnetic field or current sensor.

## 1. Introduction

A smart grid is an electrical grid that utilizes advanced technology to collect and analyze data from its various components, enhancing the power grid’s reliability, efficiency, and sustainability [1]. A smart grid system is distinguished by its use of communication networks for gathering and transmitting data, and recently they have been replacing traditional electrical grids. Smart grids offer numerous advantages over conventional grids [2,3,4,5,6,7,8]. They can be considered more reliable since they can quickly identify and isolate problems within the grid, reducing the frequency and duration of power outages. Moreover, they are more decentralized, allowing them to utilize a variety of electricity sources, including renewable energy such as solar and wind power. This decentralization makes them easier to control and more manageable. Additionally, smart grids can be more cost-effective by diminishing the need for expensive infrastructure upgrades and by optimizing electricity utilization. However, smart grids rely on sensors and wireless sensor networks (WSNs) to transmit data, which currently depend on batteries for power. As all batteries eventually fail, this can lead to data transmission failures. One potential solution to this problem is to use zero-power sensors or sensors that can also function as energy harvesters, which can provide a self-sustaining power source.

Piezoelectric cantilevers can function as both energy harvesters and sensors when integrated with a magnetic proof mass and coupled to current-carrying wires producing an AC magnetic field [9,10,11,12,13,14]. These piezoelectromagnetic structures operate by coupling the magnetic proof mass to the AC magnetic field generated from a current-flowing wire thus producing an alternating force on the cantilever. The alternating force induces stress/strain in the piezoelectric film, which transforms this mechanical strain energy into electrical energy. These devices have the potential to function both as energy harvesters [10,11,12] and as sensors [13,14], thus essentially creating a zero-power sensing WSN, which could enable data transmission without power consumption [15]. This technology has been used to fabricate both large-scale and micro-scale structures. However, it has several limitations. The cantilever resonant frequency needs to match the current resonant frequency to achieve optimal output. However, most MEMS-based devices for this application are made from silicon, which has a high elastic modulus making it difficult to design a robust low-frequency device (50/60 Hz). Using a flexible polymer-based cantilever can overcome these challenges but creating polymer piezoelectric and magnetic films is challenging and has not been achieved previously.

Researchers have developed various methods to fabricate and integrate permanent magnet proof masses, utilizing NdFeB [16,17,18,19] because of its high remanence and coercivity. Some researchers have validated macro-scale versions by using commercially purchased NdFeB magnets. Sensitivity and power density improvements have been pursued through the optimization of magnet placement and cantilever properties. In an earlier study utilizing a single wire conductor, it was indicated that optimal placement for the vertical force component occurred at a 45-degree angle to the wire. On the other hand, in recent research, it was indicated that the overall torque, including both lateral and vertical forces, also plays a crucial role, particularly as magnet size increases [16,20]. This is why comparatively small magnets are primarily affected by the vertical force, confirming earlier findings. In contrast, larger magnets will generate larger forces due to the volume expansion, which will increase the power density. It was emphasized that the cantilever dynamics were essential, with optimal angles ranging from 33 to 40 degrees depending on the stiffness of the cantilever [15]. Furthermore, longer magnets exhibit asymmetrical peaks in voltage output and magnetic field strength, which requires precise positioning to achieve sharp, narrow voltage peaks along the wire. This asymmetrical behavior was demonstrated by a previous spatial optimization study that utilized thick commercial magnets [15,21]. Research in this area agrees that accurately locating the magnet is essential for power maximization. Recent studies examined the optimal shape to broaden the sensitivity region and concluded that a trapezoidal- or triangular-shaped proof mass provided the best spatial independency [22,23], as the shape provides a more uniform force distribution on the cantilever.

Thin films have been commonly used in sensor and harvester applications. It was demonstrated that an all-thin-film magnetoelectric device on micromachined Si cantilevers reduced the clamping substrate effect [24]. Researchers optimized the interface between the magnetostrictive FeGa and piezoelectric PZT layers by using a Pt layer. Onuta et al. [22] examined the performance of all-thin-film magnetoelectric heterostructures for electromagnetic energy harvesting when located on Si cantilevers. The devices showed a maximum power of 0.7 mW/cm^3^ (RMS) at a magnetic field of 1 Oe and a resonant frequency of 3.8 kHz. Researchers are developing new methods of depositing thin film magnetostrictive materials to integrate with MEMSs [25,26]. Todaro et al. [23] suggested that piezoelectric MEMS energy harvesters fabricated using polymer thin films are cost-effective and compact microgenerators are capable of collecting energy from environmental vibrations. A three-layered polymeric composite device was constructed to operate as a sensor and harvester [24]. The device demonstrated that a multi-layer polyimide composite could be fabricated.

Piezomagnetic cantilevers have been extensively investigated as energy harvesters or current sensors for current-carrying wires [11,15,16,20,24,27]. Most of the devices investigated consist of large-scale, commercially bought components that were assembled to prove the concept or to optimize the spatial dependency. There are several MEMS-based devices [12,14,16,18,28,29,30], but each of them consists of Si-based cantilevers. These devices have demonstrated sensor accuracy < 1 mA, but they were only used in low-current applications < 1 A, due to the high stress in the Si-layer. Thus, these are not suitable for high-current applications. However, polymer-based devices could overcome this challenge.

This research focuses on the development of an all-polymer composite MEMS device, using polyimide (PI) as the base material with various composites such as Ag-PI to act as electrodes, PZT-PI as the piezoelectric material, and either NdFeB-PI or Terfenol-D–PI as the magnetic proof mass. A comparison study between NdFeB and Terfenol-D proof masses was investigated. The usage and effect of magnetic and magnetostrictive proof masses on the thin film sensor/harvester as a function of magnetic strength was performed, along with the development of a Terfenol-D–PI cantilever to act as a sensor. The authors discuss the feasibility of multilayered, multifunctional polymer composite thin film devices considering the fabrication difficulties. Finally, the authors suggest that a magnetostrictive-PI thin film could be used as a single-layer sensing device when integrated with coils. An all-polymer-based device has several potential advantages including cost and ease of large-scale manufacturing. Polymers typically have lower elastic modulus which makes a low frequency (50/60 Hz) more feasible, and polymers can handle large deflections without failure which is needed for high-current applications like power lines. The novelty of this study is in the fabrication of a multilayered all-polyimide composite device that can handle high currents while retaining high sensitivity or power density.

## 2. Materials and Methods

**Figure 1:**
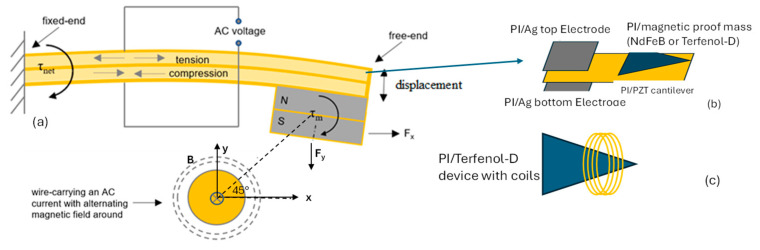
(**a**) Schematic diagram illustrating the actuation mechanism, (**b**) schematic of the four-layered all-polyimide composite cantilever structure, and (**c**) a single PI-Terfenol-D magnetostrictive film with integrated coils where τ is the torque and N and S are the poles of the magnet.

The overall concept of the piezomagnetic cantilever device and how it couples with the AC magnetic field generated from a current-carrying wire is illustrated in Figure 1. The AC magnetic field couples with the magnetic or magnetostrictive proof mass creating a vertical force and torque on the cantilever. The piezoelectric film then converts the mechanical stress/strain into an AC voltage. Figure 1b,c illustrate the various polyimide devices that were fabricated and assembled to create an all-polymer composite device. Previous papers describe the concept in more detail [12,15,16,18].

### 2.1. Fabrication of Functional Polymer Composites

PI-2611 liquid resin (HD Microsystems, Cheesquake, NJ, USA) was used as a polyimide matrix to synthesize the various composites. This polyimide was selected based on its thermal and mechanical properties. The composite films consisting of Ag-PI and PZT-PI were based on previous research [24]. Electrically conducting composites were made using 40 wt.% Ag nanoparticles (60 nm) (SkySpring Nanomaterials Inc., Houston, TX, USA) and the PI-2611 resin, which, after imidization, acted as the top and bottom electrodes for the piezoelectric film as shown in Figure 1a. The electrical conductivity was measured using a 4-point probe setup (Ossila, Sheffield, UK) to verify that the films had sufficient conductivity. The piezoelectric cantilever of the sensors/harvesters consisted of a PZT-PI composite using PZT microparticles (5 μm) (American Elements, Los Angeles, CA, USA) with a composition of 50–60 wt.%. The process for synthesizing these films was previously described in more detail [24]. The piezoelectric coefficient d_33,f_ was measured using a high-resolution (0.01 pC N^−1^) piezometer (Piezotest, PM300, Singapore).

In this study, various thin film proof masses were developed and integrated into the multilayer structure. The materials for the proof masses consisted of a PI composite using NdFeB and Terfenol-D microparticles. These two magnetic materials were selected because of their high magnetic properties (NdFeB, MQP-S-11-9 (Magnequench, Pendleton IN, USA)) and magnetostrictive (Terfenol-D (American Elements)) properties, respectively. Initially, a 40 wt.% concentration of each set of microparticles was mixed in the polyimide matrix for comparison. The magnetic property of the magnetostrictive Terfenol-D–PI composite was low. A Helmholtz coil was used to apply the magnetic field. A single copper wire with flowing current was used to further validate the concept in a real-world scenario.

**Figure 2:**
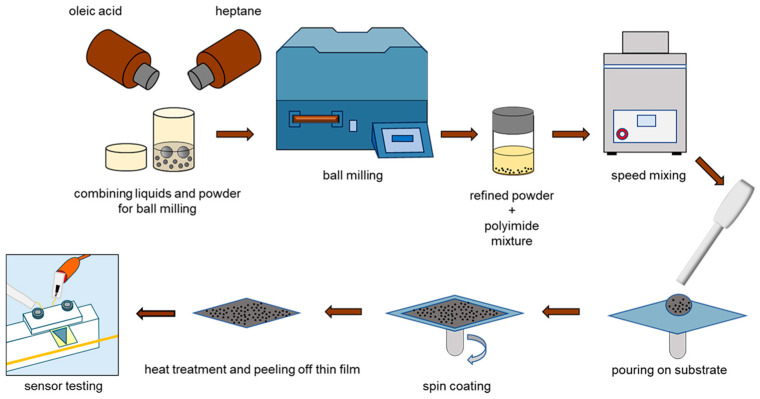
Schematic diagram illustrating manufacturing of PI composite film.

The ball milling process was used to reduce the size of Terfenol-D particles, which were around 300 µm initially, to a size of 500 nm [31]. Oleic acid (0.3 g) was used as a surfactant for the Terfenol-D powder and 10 mL heptane was used as the medium. They were poured into a zirconia ceramic vial and milled using a (Spex 8000M mixer/mill, Methcen, NJ, USA). A 5 h milling time was used with incremental 10 min breaks every 90 min. Then, the refined particles were mixed with PI-2611 using a speed mixer (Flacktek, Landrum, SC, USA) at 1200, 2500, and 3000 rpm, respectively. Finally, this was poured onto a Si/glass substrate and spin coated. The PI composites were then imidized at 350 °C for 30 min with a ramp rate of 5 °C. The films were then patterned using dry etching to form the shape and peeled off the substrate. Figure 2 demonstrates the whole procedure for the fabrication of Terfenol-D–PI thin films. The NdFeB-PI samples were prepared using the same process. After imidization, the films were magnetized in an impulse magnetizer at 2.7 T.

Using synthesized thin films, multi-layer sensors were fabricated by combining the functional layers together. The layers were then bonded together under a microscope for alignment. The bonding was performed using compression force and then the layers were wrapped in a single layer of polyimide tape to hold them together. Resonant frequencies for each device, which were between 50 and 60 Hz, were measured on a vibration shaker and the frequency of the magnetic field was set to match each device’s resonant frequency to maximize amplitude. The frequency of each device varied slightly due to the concentration of particles which affected the overall density.

Initially, 40 wt.% concentration Terfenol-D–PI and NdFeB-PI were fabricated and used as proof mass. Based on the results, various amounts of the NdFeB proof mass samples and Terfenol-D samples were fabricated and tested separately. NdFeB-PI samples with various concentrations ranging from 40 wt.% to 70 wt.% were fabricated and experimentally tested to determine the effects of varying magnetic composition. The Terfenol-D–PI samples’ compositions ranged from 10 wt.% to 40 wt.%, as above 40% the polymer had difficulty curing properly.

### 2.2. Experimental Characterization

**Figure 3:**
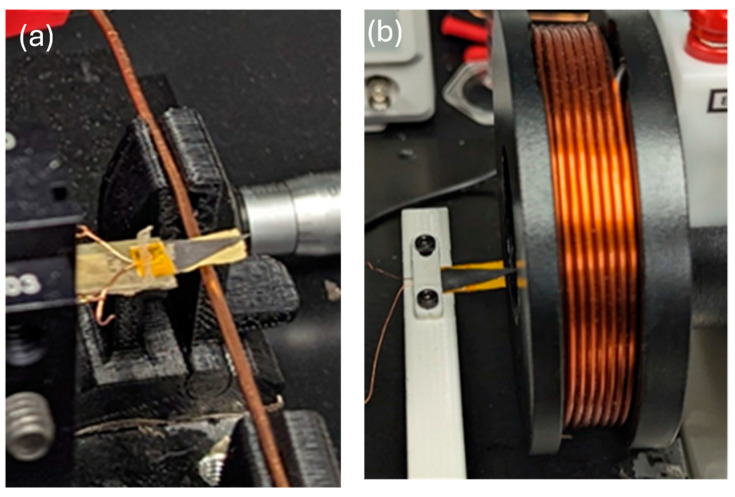
Experimental testing: (**a**) single conducting wire and (**b**) Helmholtz coil setup.

A custom-made experimental setup was developed as shown in Figure 3. The sensor/harvester device was assembled on a micro-manipulator with an x-y translation stage, the maximum resolution of which is 0.635 mm. The device was placed near a single-current bare Cu wire or inside the Helmholtz coil as demonstrated in Figure 3.

A single copper wire was utilized for the multilayered sensor/harvester with an NdFeB-PI proof mass to illustrate potential application in a real-world scenario. However, for more controlled testing, the Helmholtz coil provides a higher magnetic field and was used to compare the two different proof masses.

A pulsed biphasic DC current (Chroma 62006P-30-80, Foothill Ranch, CA, USA) was used in both setups to mimic an AC force on the cantilever which included a controllable electronic load (Chroma 63004-150-60, Foothill Ranch, CA, USA). The frequency of the current was adjusted to match each cantilever’s resonant frequency. The initial testing was performed at 1 A, but the current was varied later to demonstrate the impacts of varying currents. The voltage (V_pp_) output generated by the cantilever was measured using an oscilloscope. Two 26-gauge wires were bonded to the electrodes using conducting epoxy which was then connected to the oscilloscope to monitor the voltage output.

**Figure 4:**
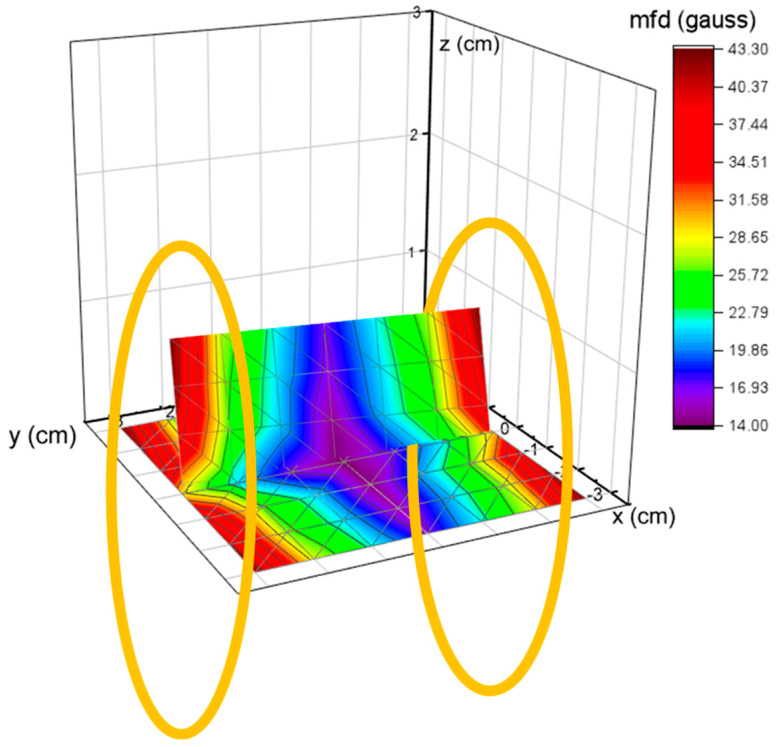
Magnetic field strength graph between Helmholtz coils (yellow circles) based on gaussmeter measurements.

The magnetic flux densities, as a function of location on the Helmholtz setup where the coils were spaced 1 cm apart, were measured by using an (Alphalab GM-2, Salt Lake City, UT, USA) gaussmeter, and the magnetic field strength map was constructed as shown in Figure 4. The Helmholtz coils are represented by orange circles. The magnetic flux density values increase near the coils. The points were selected on the x-y and y-z planes, with their intersection line passing through the centers of the Helmholtz coils. The highest magnetic flux densities were observed near the coils as shown in the graphs. Therefore, the position for the thin film sensor tests was decided to be 1 cm from the bottom of a coil.

## 3. Results and Discussion

### 3.1. Proof Mass Comparison

Multilayered thin film polyimide sensors with varying proof masses (NdFeB-PI and Terfenol-D–PI) were compared. The electrical conductivity of the 40 wt.% Ag-PI films was 6 kS/m. The PZT-PI thin film had a d_33,f_ value 6 pC/N which is similar to previous studies [24]. In addition, the magnetic strengths of NdFeB-PI and Terfenol-D–PI composite thin films were 0.34 and 0.033 T, respectively, which is similar to other NdFeB composites [32,33]. Considering the remanence value of the NdFeB powder was 0.745 T, the estimated fill factor was 45% which is similar to the 40% concentration used.

**Figure 5:**
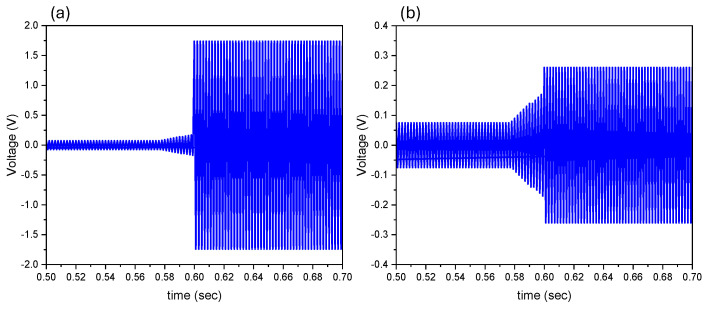
Voltage output of the 40 wt.% multilayered sensor using 1 A in Helmholtz coil: (**a**) NdFeB-PI magnetic proof mass and (**b**) Terfenol-D–PI magnetostrictive proof mass.

Figure 5 demonstrates the peak-to-peak voltages obtained from the sensors before and after the applied magnetic field. The sensor with the NdFeB composite thin film proof mass generated an average V_pp_ of 3.48 V, while the Terfenol-D–PI proof mass device produced an average V_pp_ of 0.52 V. This was expected since the NdFeB-PI composite had an order of magnitude higher remanence value. It can be concluded that the device with the NdFeB-PI proof mass will generate higher voltage output and thus be better for energy harvesting applications. Although the use of Terfenol-D–PI had a lower voltage output, the concept was validated, and since Terfenol-D is a magnetostrictive material it can be actuated under a magnetic field to function as a magnetic field sensor. The voltage output was significantly lower than other Si-based MEMS devices, which is to be expected due to the lower piezoelectric properties and lower elastic modulus of polyimide compared to Si.

### 3.2. Sensors with Magnetic Proof Mass

The NdFeB-PI proof mass depends on the thickness of the film and the fill factor or concentration of the NdFeB in the film. The thickness of a composite film depends on the spin speed at which it is deposited and the viscosity of the liquid which depends on the concentration. The spin coating speeds varied from 0.5 to 1 krpm, which resulted in a film thickness of 15–25 μm depending on the concentration of nanoparticles. The concentration of NdFeB varied between 60 and 70 wt.% as these were the highest concentration values that could be used and still maintain a polymer film. The films were patterned to be a triangle with dimensions of 15 × 8 mm, and a short rectangle of 4 × 8 mm.

The gaussmeter with a resolution of 1 × 10^−6^ T was used to assess the magnetic strength at various positions across the magnetic thin films. The average results are shown in Table 1. The thicker magnets with a lower spin speed of 0.5 krpm and a concentration of 70 wt.% demonstrated higher magnetic properties, as expected. The shorter rectangular magnet had higher properties than the other shapes probably due to the distribution of magnetic particles within the film. In both scenarios, it is evident that a higher particle count increases the magnetic strength.

**Figure 6:**
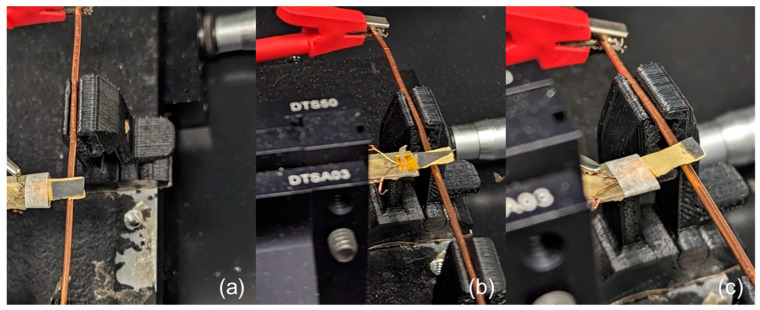
Experimental setup with various shaped proof masses: (**a**) long rectangle, (**b**) triangle, and (**c**) short rectangle, coupled to a single Cu wire to determine spatial resolution.

After the sensor/harvester devices were fabricated, they were connected to the custom-made setup as shown in Figure 6. The spatial position of the devices was changed from −6.35 mm to 6.35 mm, where the 0 position was the center position of the magnet proof mass. The peak-to-peak voltage values were recorded by the oscilloscope for each position and the results were compared to previous results that used bulk NdFeB magnets of similar shape and dimensions [20].

**Figure 7:**
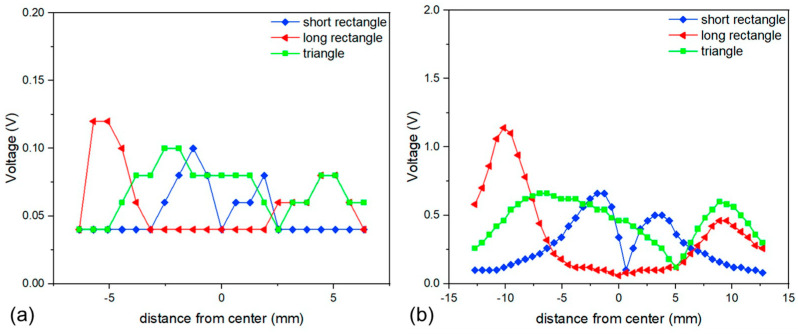
Spatial resolution comparison of (**a**) thin film devices versus (**b**) bulk devices [20].

As previous studies indicated, the position and shape of the magnet are critical as they determine the torque applied to the cantilever. Results illustrating the voltage output as a function of spatial orientation to the wire are shown in Figure 7. Although the voltage values obtained for the all-polymer composite devices were an order of magnitude lower than the results using bulk NdFeB magnets, the general shape and spatial dependencies are similar. The voltage output response was expected as the magnetic properties of the composite films were an order of magnitude lower than typical sintered NdFeB magnets (1.4 T). Therefore, the force produced on the device would be lower. The triangular thin film magnet proof mass showed the broadest sensitivity region for the thin film sensor which was in agreement with previous work [20]. This demonstrates that a triangular magnetic proof mass was better for sensing applications as the output voltage was not dependent on an exact spatial location in relation to the wire.

**Figure 8:**
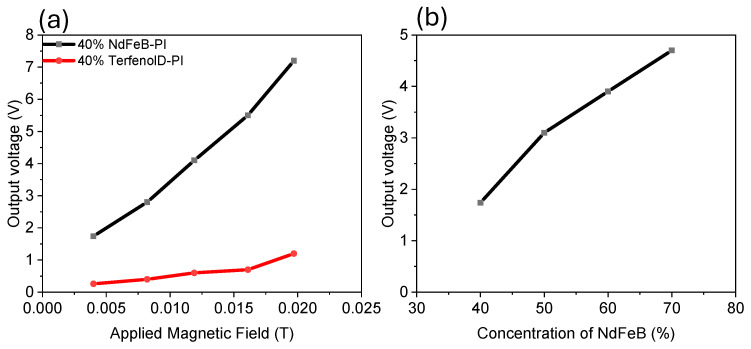
Voltage output of piezoelectric composite with magnetic proof mass (**a**) as a function of applied magnetic field and (**b**) as a function of NdFeB composition.

The output voltage was dependent on the magnetic properties of the film as well as the applied magnetic field. Figure 8 illustrates the output voltage as a function of the applied magnetic field using the Helmholtz coil. It was shown that as the applied magnetic field increased so did the output voltage, and that the NdFeB-PI proof mass device had significantly higher voltage. The voltage as a function of NdFeB concentration is also illustrated in Figure 8b, and as expected the output voltage increased with increasing NdFeB concentration.

### 3.3. Magnetostrictive Sensors

**Figure 9:**
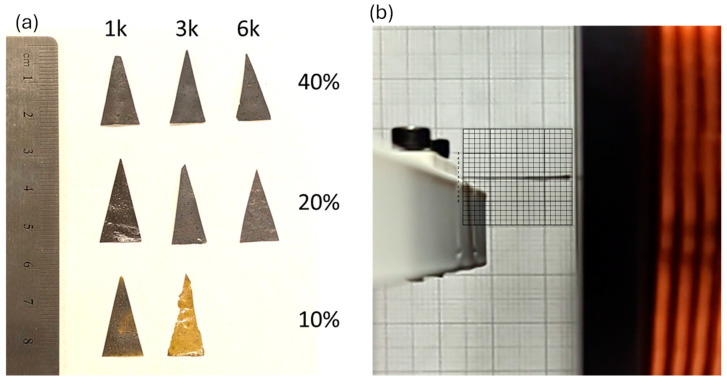
(**a**) Triangular-shaped magnetostrictive films with different Terfenol-D concentrations and spin coating speeds (1k, 3k, and 6k), (**b**) the experimental setup to measure tip displacement under various magnetic fields.

Since the magnetostrictive thin film (Terfenol-D–PI) composite did not demonstrate high voltage output when used as a proof mass in the multi-layered sensor, it was decided to investigate using the composite material as the cantilever structure similar to the PZT-PI composite film. Triangular-shaped composites with varying spin speeds and varying concentrations of Terfenol-D are shown in Figure 9. The individual devices were placed in the Helmholtz coil and their displacement was measured using a high-speed camera and grid paper with 500 μm resolution lines as shown in Figure 9b.

**Figure 10:**
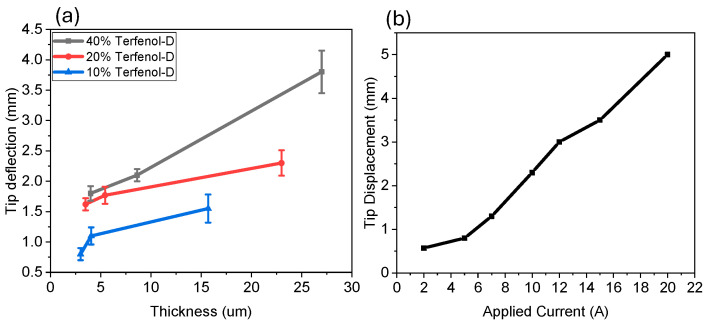
Tip deflection of triangular-shaped Terfenol-D–PI films with (**a**) varying thickness and composition and (**b**) 20% Terfenol with 23 μm thickness with varying applied current.

Previous experiments using magnetostrictive materials were conducted to illustrate the Joule effect, which is essentially the inverse of the Villari effect [34,35], which illustrates that a magnetostrictive material wrapped in an electrically conducting coil can convert mechanical movement into voltage/current through the coil. A Helmholtz coil was used to provide a sufficient magnetic field for the system which would allow the magnetostrictive film to elongate and compress, thus generating a current/voltage. The average maximum displacement of *n* = 20 runs is shown in Figure 10. The results illustrate that the more concentrated Terfenol-D composites and thicker films both resulted in higher tip deflection. Although thinner films require less force to deflect the cantilever, the fact that there are more Terfenol-D particles in the thicker films resulted in higher forces causing higher deflection. In addition, tip deflection was also significantly impacted by applied magnetic field as illustrated in Figure 10b.

**Figure 11:**
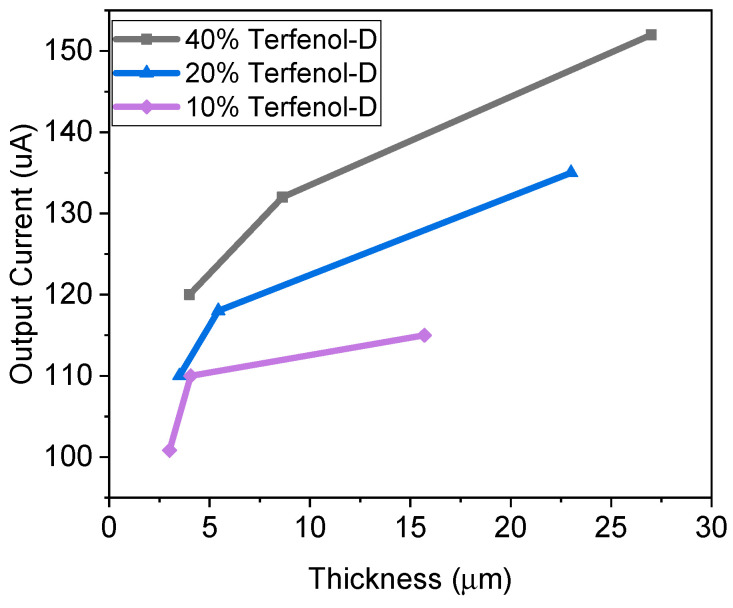
Current output of Terfenol-D–PI composite film with a magnetic field of 0.012 T.

The Villari effect on the triangular magnetostrictive polyimide composite samples was investigated. A 50 μm diameter Cu wire was used to create a coil having 200 turns which was placed in between the Helmholtz coils and connected to a multimeter. When there was no device inside the coil, the current measured was low (approximately 9.3 µA) due to small vibration-based fluctuations. Then, the Terfenol-D–PI composite films were placed inside the coil and the current through the Cu coils was monitored and illustrated a significant increase as shown in Figure 11. The results demonstrate that the more concentrated and thicker films produced higher current, as expected, as these had higher magnetostrictive properties. The resulting values were in parallel with tip deflection values as the strain was proportional to the magnetic field created. This demonstrates that a simple magnetostrictive single-layer film of Terfenol-D–PI composite could be used as a sensor or energy harvester.

## 4. Conclusions

This study demonstrated the successful fabrication of two types of polymer-based electromagnetic thin film sensors. One device consisted of a four-layer polymer composite structure using Ag-PI, PZT-PI, and either NdFeB-PI or Terfenol-D–PI. The structure using NdFeB-PI proof mass demonstrated significantly high output voltage in the presence of a magnetic field and could be used to sense current or magnetic fields and could also be used as an energy harvester to create a zero-power system as previously demonstrated.

The second device consists of a single layer of Terfenol-D–PI which, when used in combination with a coil, demonstrated a current output in the presence of a magnetic field. This device was simpler to manufacture as it only had one layer as opposed to four layers with different composite materials. The use of magnetic and magnetostrictive nano-/microparticles in polymer composites has significant advantages such as cost of manufacturing and ease of manufacturing compared to bulk material integration and has potential applications in MEMS sensing and energy harvesting.

The all-polymer device demonstrated a larger tip displacement (mm range) compared to previous Si-based MEMS devices which illustrated displacements in the μm range [12]. The voltage output and power were lower for the all-polymer device due to a reduced elastic modulus in a polymer compared to Si and the PI/PZT and PI/magnetic devices have lower functional properties than pure films due to the fill factor.

## Figures and Tables

**Table 1:** Magnetic Properties of Composite Thin Films.

Composite Composition	Triangle (T)	Long Rectangle (T)	Short Rectangle (T)
60% NdFeB + 2611 1k	0.45	0.442	0.48
60% NdFeB + 2611 0.5k	0.492	0.473	0.789
70% NdFeB + 2611 1k	0.53	0.489	0.651
70% NdFeB + 2611 0.5k	0.838	0.727	0.91

## Data Availability

Data are contained within the article.
